# Empiric Treatment of Focal Organizing Pneumonia in a Patient with a Low-Risk Lung Mass

**DOI:** 10.1155/2013/340202

**Published:** 2013-07-30

**Authors:** Mir Alikhan, Srihari Veeraraghavan

**Affiliations:** Division of Pulmonary, Allergy and Critical Care, Department of Medicine, Emory University School of Medicine, 615 Michael Street, Suite 205, Atlanta, GA 30322, USA

## Abstract

The authors present a case of a 57-year-old man presenting with a solitary lung mass. Transbronchial biopsy showed an organizing pneumonia pattern. A therapeutic trial of corticosteroids resulted in complete resolution avoiding surgery. The authors discuss the diagnosis of focal organizing pneumonia without surgical resection.

## 1. Case Presentation

A 57-year-old male was referred to the pulmonary clinic for an abnormal computed tomography (CT) scan. The patient initially presented to his primary care physician with two weeks of dry cough, fatigue, and body aches. He underwent a chest radiograph and CT scan of the chest, which showed a mass in the right middle lobe. He denied any symptoms of shortness of breath, wheezing, hemoptysis, fever, or weight loss.

The patient's past medical history is significant for hypertension, hyperlipidemia, and gastroesophageal reflux disease. He has never smoked cigarettes and denied use of alcohol or illicit drugs. The patient did not have any significant medication, environmental, or occupational exposures. He also did not have any pets in his home. He denied history of significant travel or ill contacts. His family history was insignificant for any respiratory diseases or malignancy.

On physical examination, the patient was a well-appearing, middle-aged Caucasian male. He was in no respiratory distress and had normal vital signs. His pulmonary exam demonstrated clear bilateral breath sounds without rales or rhonchi, and the remainder of his exam was otherwise unremarkable.

Laboratory data obtained prior to his initial evaluation demonstrated a normal chemistry panel except for an elevated creatinine (1.45 mg/dL). Complete blood count demonstrated a mild leukocytosis (11.9 × 10^3^/*μ*L) and normocytic anemia (10.9 g/dL). No other serum analysis was performed.

**Figure 1:**
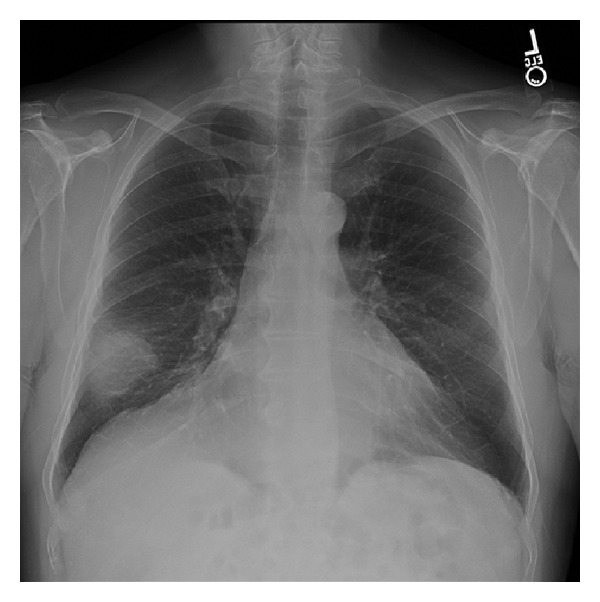
Chest radiograph demonstrating a large mass in the right middle lobe.

Pulmonary function tests revealed normal spirometry, lung volumes, and diffusion capacity. Chest radiograph demonstrated a mass in the lateral segment of the right middle lobe with an otherwise normal appearance of the lung parenchyma (Figure 1).

**Figure 2:**
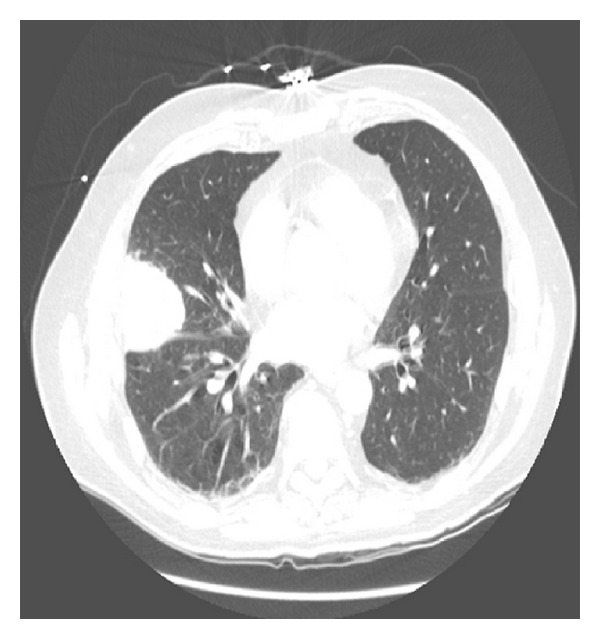
Chest CT scan demonstrating a large, rounded mass in the periphery of the right middle lobe.

CT scan of the chest with intravenous contrast was also performed. This was most significant for a right middle lobe mass measuring 4.4 cm × 4.8 cm (Figure 2). There were no pathologically enlarged lymph nodes or pulmonary embolus. The remainder of the lung parenchyma was normal in appearance.

Given the combination of findings, fluoroscopic guided bronchoscopy was performed. Endobronchial ultrasound (EBUS) did not show any pathologically enlarged lymph nodes. Bronchoalveolar lavage (BAL) was performed in the right middle lobe. Transbronchial biopsy of the right middle lobe mass was also done.

**Figure 3:**
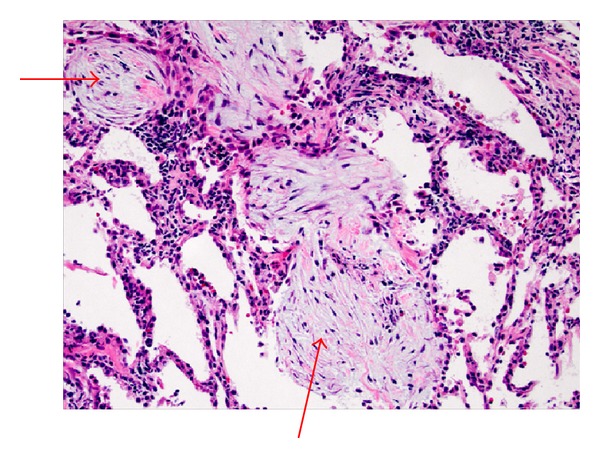
Hematoxylin & eosin stain demonstrating the hallmark intra-alveolar buds of granulation tissue that are characteristic of organizing pneumonia.

Histological examination of the specimen showed features of organizing pneumonia including intra-alveolar buds of granulation tissue (Figure 3). There was no evidence of any malignancy and special stains for fungal elements were negative. BAL cell counts were not obtained. However, BAL fluid and tissue cultures were negative for bacterial, mycobacterial, and fungal pathogens.

**Figure 4:**
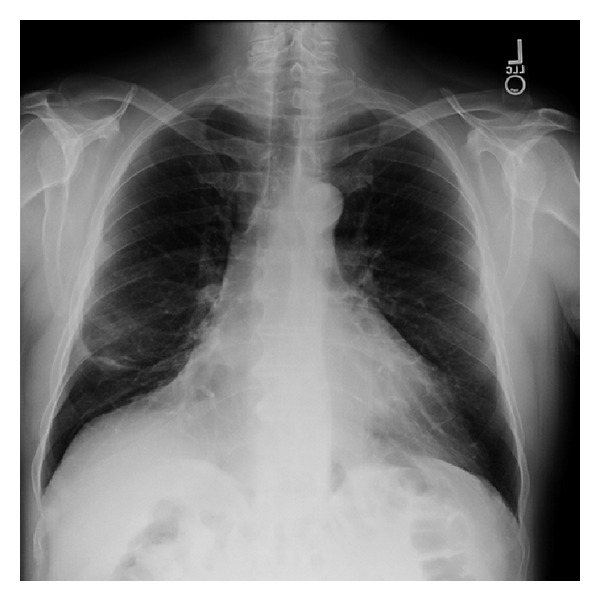
Chest radiograph demonstrating near-complete resolution of the right middle lobe mass after treatment with corticosteroids.

A presumptive diagnosis of focal cryptogenic organizing pneumonia was made and the patient was started on prednisone at 40 mg per day. He was seen three weeks later with complete resolution of his symptoms and a near complete resolution of the lesion on the chest radiograph (Figure 4). He continued systemic corticosteroid therapy with a slow taper over the next five months. Upon follow-up the patient was asymptomatic and his steroid therapy was subsequently stopped. The patient was seen again nearly fourteen months after the initial diagnosis. He was doing well and had no signs or symptoms of recurrence. He continues to be followed periodically to ensure stability.

## 2. Discussion

Organizing pneumonia is defined histopathologically by the presence of buds of granulation tissue in the distal air spaces [1]. This pathological pattern is the hallmark of the inflammatory lung disease of unknown cause with distinctive radiological and clinical features called cryptogenic organizing pneumonia (COP). COP is a distinct entity among the group of idiopathic interstitial pneumonias and usually presents with a subacute time course and nonspecific clinical features. Imaging characteristics include multiple bilateral patchy peripheral alveolar opacities, which may have varying densities including ground glass changes or consolidation. Less common radiological patterns include diffuse bilateral infiltrates and focal solitary lesions. Video assisted thoracoscopic lung biopsy is the recommended method for diagnosing COP.

Focal solitary COP is a rare entity which may present with imaging similar to malignant lesions like bronchogenic carcinoma [2, 3]. In most case series of focal COP the diagnosis was made after a surgical resection of the offending lesion. Though the ATS/ERS consensus classification on idiopathic interstitial pneumonias suggest that a diagnosis of COP may be confirmed by transbronchial biopsy in the appropriate clinical setting, many experts believe that a surgical lung biopsy is needed for confirmation as such pathological changes can occur around vasculitis, eosinophilic pneumonia, nonspecific interstitial pneumonia, hypersensitivity pneumonitis, and malignancy [1, 4]. Kohno et al. described 18 patients with focal COP among which 10 were diagnosed based on transbronchial biopsy. Two out of the 18 patients were reported to show complete resolution of the mass on imaging though the treatment administered and duration of follow-up were unclear [3].

Our patient presented with nonspecific symptoms and a CT scan, which was highly suspicious for lung cancer. Transbronchial biopsy showed classic organizing pneumonia and EBUS did not show any enlarged lymph nodes. He was a non-smoker with no exposure history or family history of lung cancer. While a malignant process including bronchogenic carcinoma or less likely primary pulmonary lymphoma was still a possibility, we elected to treat him empirically with steroids for a defined period of time. In view of his low risk status for malignancy we felt that a three-week delay would be unlikely to negatively impact his outcome. Three weeks after starting prednisone at 40 mg per day the patient was seen in clinic with complete resolution of his symptoms and near complete resolution of the mass on chest radiograph (Figure 4). 

We suggest that in suspected cases of focal COP with the appropriate clinical setting, a trial of therapy with corticosteroids for two to four weeks may help avoid an invasive procedure.
